# Supplementary material for: The Neglected Tropical Disease Non-Governmental Organization Network: The role of a global health network in an evolving global health landscape
Source: PLOS Glob Public Health. 2025 Jan 31;5(1):e0004078. doi: 10.1371/journal.pgph.0004078 (PMC11785335; doi:10.1371/journal.pgph.0004078)
Supplement: Attachment — Submitted filename: Response to Reviewers.docx [file pgph.0004078.s002.docx]

3465, Scotts Mill Run

Duluth GA 30096

Bram Wispelwey, MD, MS, MPH

Academic Editor

PLOS Global Public Health

19 November 2024

Dear Dr. Wispelwey,

Thank you for your email dated 9 October 2024, and for the opportunity to revise our manuscript. My co-authors and I are pleased to submit our revised manuscript titled, “The neglected tropical disease non-governmental organization network: the role of a global health network in an evolving global health landscape,” for your reconsideration for publication in PLOS Global Public Health.

We thank you and the reviewers for your time and effort in reviewing our manuscript. The feedback has been invaluable in improving the content and presentation of the paper.

We have revised our manuscript according to the reviewers’ comments. The changes are highlighted in the attached manuscript, and our point-by-point responses are given after the word “Response:” below.

Additionally, we have noted your emphasis to reflect on the question of denominator in Reviewer 2’s first comment. We have edited the wording to be more precise in our finding, as evident in lines 30, 31, 205, and 206. We have also addressed this finding further in lines 179 – 184.

-

**Responses to Reviewer 1:**

**Comment 1:**

This is a well-written paper on a very timely and important topic. I appreciated the rationale for undertaking the organizational analysis and agree with the authors' call to action for other networks/organization to undertake a similar activity. In addition to these overall comments, I have the following comments/questions:

Given that some NGOs/organizations could consist of many programs, how were such organizations counted and treated in the analysis? For example, I am thinking of an organization such as the Task Force for Global Health, which is an organization itself but also consists of 16-17 programs - many of which have their own mission, objectives, target population of focus, and disease/domains of focus. Each program also participates in different coalitions and has different networks. Was the TFGH counted as one of the 91organizations in the analysis or were each of its programs counted? Could the authors comment on this and explain how either approach could impact the results?

**Response:**

We thank reviewer 1 for their overall comments.

As the reviewer rightly points out, some international NGOs serve as umbrella organizations hosting several NTD programs. Reviewer 1 took the example of The Task Force for Global Health (TFGH), which is an umbrella organization hosting several NTD programs, such as the NTD Support Center (now called the Coalition for Operational Research on NTDs), the International Trachoma Initiative (ITI), and the Mectizan Donation Program (MDP), to name a few. We counted and considered the members based on their registration with the NNN as a member, i.e., the TFGH was counted as a separate member organization because it was registered as such; the ITI and MDP were also counted separately as members because they were registered as such. For the purposes of this analysis, we did not group these “programs” into one parent organization because we wanted to stay true to the membership list.

Upon further analysis and review of the membership list, the Task Force for Global Health appears to be an exception in that both the umbrella organization and its members are listed separately.

While considering organizations like the TFGH and its programs as separate entities (as they chose to be listed in the NNN membership) may serve as double counting of the reach of U.S. based organizations (in this instance, continuing with the TFGH example), we do not expect the overall findings and recommendations to be affected by this approach. Moreover, the programs at the TFGH have made individual and separate contributions to the NNN by way of annual sponsorships of the NNN conference and stand apart in their identities. We believe it would have been a misrepresentation to group such programs into one umbrella.

The NNN is currently undertaking a strategic review where exactly such topics will be addressed, i.e., how to treat programs and their umbrella organizations within the membership.

The rationale for this analysis and paper also lies in signalling to the wider global health community, the intricacies and nuances of the NTD landscape.

**Comment 2:**

Given the growing interest in making health campaigns, including those campaigns related to NTDs, more collaborative and possibly even include co-delivery of interventions, perhaps the authors would want to discuss how NNN member organizations that address only one NTD might model increased collaboration themselves.

**Response:**

We thank Reviewer 1 for this excellent suggestion.

We have incorporated this suggestion under point number 4 (lines 295-301) in the Conclusion section of the manuscript.

We would also like to note that one characteristic of NTD programs is the protective nature of their funding resources. These resources are typically lean and need be utilized vertically to retain donor support. While the value of co-delivery of programs has been demonstrated in some instances, it's been our experience that organizations often find it challenging to pool resources for integrated NTD campaigns.

**Responses to Reviewer 2:**

**Comment 1:**

I commend the authors for their efforts in conducting this project. Overall, the paper is well written. I have a few comments.

The authors identify the following "the African region receives nearly twice the support compared to other regions" as a key finding in the analysis, but they do not devote any discussion to this finding. What are the implications of the African region receiving nearly twice the support of other countries? Are other regions being neglected or is the higher proportion of support in the African region backed by data on the concentration of NTDs?

**Response:**

We thank Reviewer 2 for their overall comments.

In response to comment 1,

We appreciate Reviewer 2’s request for additional discussion on the finding that the African region receives nearly twice the support compared to other regions.

We have edited the wording to be more precise in our finding, as evident in lines 30, 31, 211, and 212.

We have also addressed this finding further in lines 182 – 187 with the following addition:

“While countries in Africa bear the highest burden of some diseases (i.e., trachoma in Ethiopia or onchocerciasis in the Democratic Republic of the Congo), collectively, and based on the most recent WHO global report on NTDs 2024, 51.4% of the 1.62 billion people requiring interventions for NTDs were in the WHO South-East Asia Region, followed by the African Region at 35.8% (13). This finding may point to the need for increased engagement with potential or new members active in NTD elimination in the South-East Asia Region.”

**Comment 2:**

The figures in the paper are blurry. Figure 4 in particular is illegible.

**Response:**

We have uploaded clearer figures with this revision. The revised images have passed the Preflight Analysis and Conversion Engine (PACE) digital diagnostic tool requirements.

**Comment 3:**

Line 84: the first phrase is redundant with information presented in line 43 in the introduction.

**Response:**

We have edited line 84 to address the redundancy. Please note that this edit now appears in line 91.

**Comment 4:**

Line 178 - numbers less than 10 should be spelled out.

**Response:**

We have edited line 178-181 accordingly. Please note that these edits now appear in lines 194 -195. We have also incorporated this comment in line 165.

**Comment 5:**

I recommend editing to ensure consistent formatting of the paragraphs and lists.

**Response:**

We have edited the manuscript to ensure consistent formatting of the paragraphs and lists.

-

All authors have read and approved the changes made to the manuscript. We hope that the revised paper is now suitable for inclusion in PLOS Global Public Health, and we look forward to hearing from you.

Yours sincerely,

Girija Sankar

Head of NTDs

CBM, Christian Blind Mission

Cell: +1 404 477 8320

Email: girija.sankar@cbm.org
